# Supplementary material for: Role of Homeobox A1 in Airway Epithelial Generation from Human Airway Basal Cells
Source: Cells. 2025 Apr 5;14(7):549. doi: 10.3390/cells14070549 (PMC11989199; doi:10.3390/cells14070549)
Supplement: Supplementary file 1 [file cells-14-00549-s001.zip › cells-3478515-supplementary.pdf]

Supplemental Material

Role of homeobox A1 in airway epithelial regeneration from human airway basal cells

Mohsen Tabasi[1,2], Nathaniel Chen[1], and Umadevi Sajjan[1,2,3,4]*

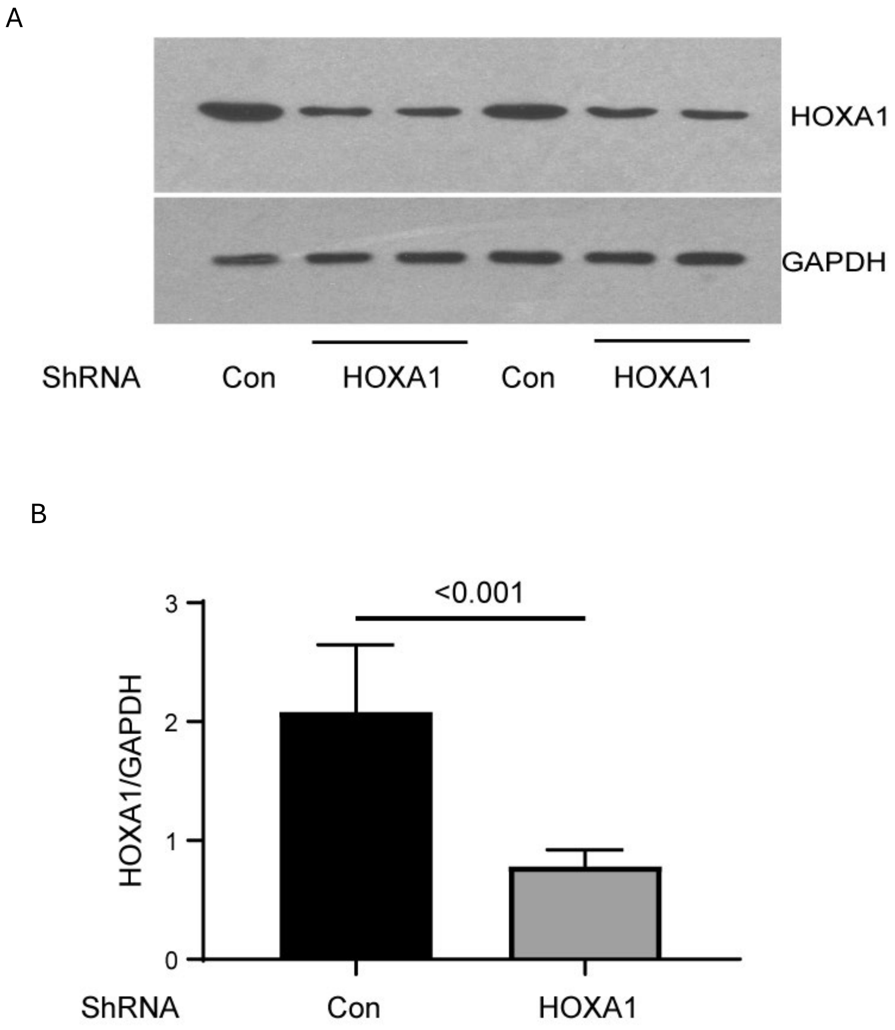

Figure S1. Western blot analysis confirms knockdown of HOXA1 by gene-specific shRNA. Normal airway basal cells transduced with HOXA1 or non-targeting shRNA expressing lentivector were cultured in transwells and grown until they reached 70 to 80% confluency. Cells were then treated with 1 μg/ml doxycycline to induce HOXA1 knockdown, incubated for another 48 to 72 h or until the cells reached confluency. A. Total protein was isolated and equal amounts of protein was subjected to Western blot analysis with HOXA1 antibody. The blot was stripped and reprobed with antibody to GAPDH. The image represents results from 2 independent experiments. B. Signal intensities of HOXA1 and GAPDH bands were measured by imageJ and presented as change over GAPDH. Results represent mean ± SEM (n=2 to 4; t test).

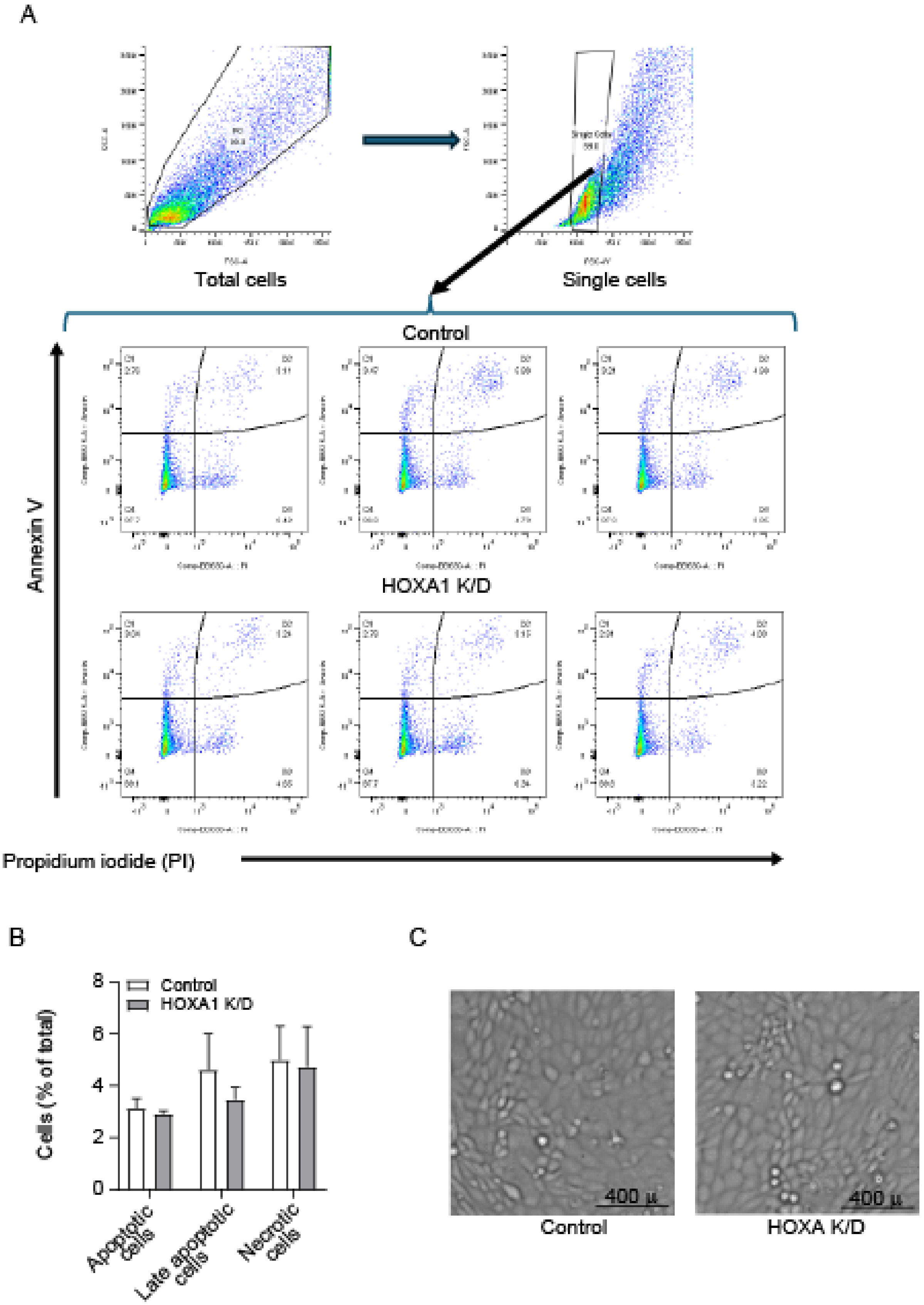
A
Total cells
Single cells
Control
HOXA1 K/D
Annexin V
Propidium iodide (PI)
B
Control
HOXA1 K/D
Cells (% of total)
8
6
4
2
0
Apoptotic cells
Late apoptotic cells
Necrotic cells
C
400 µ
400 µ
Control
HOXA K/D

Figure S2. Knockdown of HOXA1 in confluent monolayers of airway basal cells do not cause cell death. Normal airway basal cells transduced with HOXA1 or non-targeting shRNA expressing lentivector were cultured in transwells and grown until they reached 70 to 80% confluency. Cells were then treated with 1 μg/ml doxycycline to induce HOXA1 knockdown, incubated for another 48 to 72 h or until the cells reached confluency. A and B. The cells were harvested and subjected to flow cytometry with Annexin V apoptosis kit. Panel A represent gating strategy showing apoptotic (upper left quadrant), late apoptotic (upper right quadrant), necrotic (lower right quadrant) and live (lower left quadrant) cells. B. Quantification of apoptotic, late apoptotic and necrotic cells derived from flow cytometry and represents mean ± S.D. (n=3). C. Cells in the transwells were images under phase contrast microscopy. Representative 3 cell cultures.

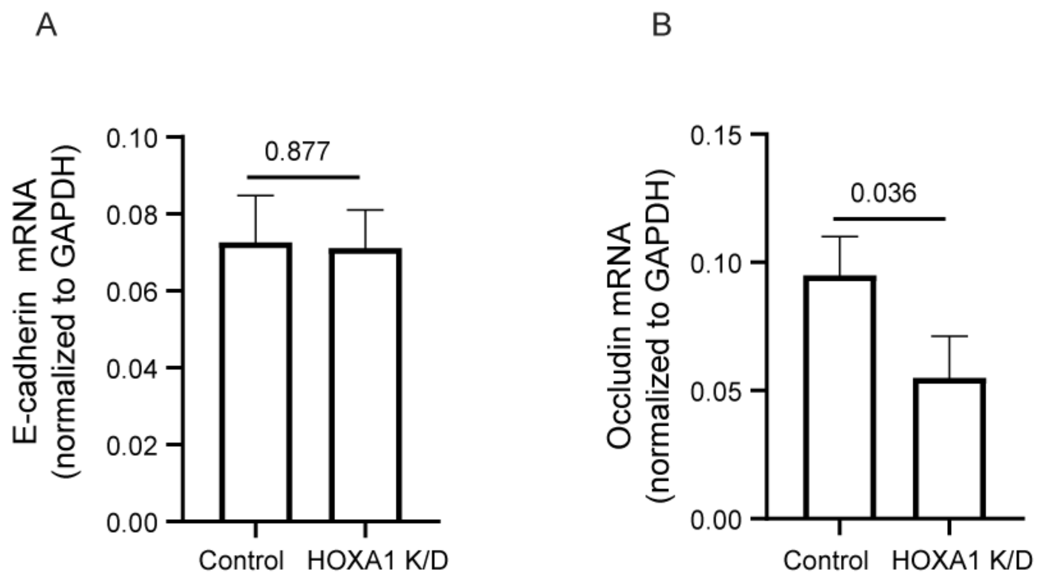

Figure S3. HOXA1 K/D cells show reduction in the expression of occludin mRNA, but not E-cadherin. Control and HOXA1 K/D cell cultures were cultured at ALI for two weeks and then the total RNA was isolated. cDNA was synthesized from total RNA and subject to gene specific probe based PCR and the expression of A. E-cadherin and B. occludin were normalized to house keeping gene GAPDH. Data is presented as mean ± S.D. calculated from 3 independent experiments (n=3, unpaired t test).
